# 超高效液相色谱法测定聚乙烯类食品接触材料中8种添加剂

**DOI:** 10.3724/SP.J.1123.2020.12002

**Published:** 2021-05-08

**Authors:** Yun LING, Jingbo BI, Wei YONG, Meiyi YAO, Yujia ZHANG, Feng ZHANG

**Affiliations:** 1.中国检验检疫科学研究院, 北京 100176; 1. Chinese Academy of Inspection and Quarantine, Beijing 100176, China; 2.中国医科大学, 辽宁 沈阳 110122; 2. China Medical University, Shenyang 110122, China

**Keywords:** 超高效液相色谱, 聚乙烯, 添加剂, 食品接触材料, ultrahigh-performance liquid chromatography (UPLC), polyethylene (PE), additive, food contact material

## Abstract

食品接触材料中添加剂残留量的测定为食品接触材料从源头进行安全监管具有重要意义。然而目前的大多数研究只针对食品接触材料中有害物迁移量的测定,对于食品接触材料中有害物含量的测定方法仅局限于残留单体、低聚体、重金属,以及邻苯二甲酸酯类、双酚类化合物等环境污染物,对食品接触材料中添加剂残留量的测定较少。该研究系统地优化了样品前处理过程及仪器分析中影响8种添加剂分析准确度与响应灵敏度的各主要因素,建立了超高效液相色谱同时测定聚乙烯材料中8种添加剂的定量分析方法。聚乙烯样品冷冻研磨后,取2.0 g样品采用甲苯作为萃取溶剂,80 ℃, 10.34~11.72 MPa (1500~1700 psi)下对其进行加速溶剂萃取,取10 mL上清液,氮气吹干后用10 mL初始流动相(甲醇-水,7∶3, v/v)定容。采用ACQUITY UPLC BEH C_8_色谱柱(100 mm×2.1 mm, 1.7 μm)进行分离,柱温30 ℃,进样量5 μL,以乙腈和水作为流动相进行梯度洗脱,流速0.3 mL/min,二极管阵列检测器(DAD)在210~400 nm范围内扫描,230、250、280、330 nm监测,外标法定量。8种目标物在0.2~10 μg/mL质量浓度范围内线性关系良好,相关系数(*R* ^2^)>0.999。空白聚乙烯样品添加含量为0.05%时,加标回收率在83.8%~103.4%之间,RSD在0.14%~7.86%之间。对于含量为0.2%~0.9%之间的质控样品,8种目标物的平均回收率在63.5%~118.5%之间,RSD在4.61%~15.6%之间。8种目标物的定量限为0.02%。应用该方法测定10份市售聚乙烯食品包装袋和手套,其中6份样品均检测出含有亚磷酸三(2,4-二叔丁苯基)酯(抗氧剂168),含量为0.02%~0.07%,均小于GB 9685-2016规定的聚乙烯类食品接触材料中抗氧剂168的最大使用量(0.2%)。该方法能够满足聚乙烯类产品中8种添加剂的分析要求,可用于食品接触材料风险监测。

## Abstract (en)

Measurement of additive residues in food contact materials is important for safety monitoring at the initial stage. Most of the current studies focus on the determination of the migration amounts of chemical hazards from food contact materials into food simulants. Studies on chemical hazard residues in food contact materials are limited to monomers, oligomers, heavy metals, phthalic acid esters, and biphenols, which are known environmental pollutants. Only a few studies have investigated analysis methods for additive residues in food contact materials. In this study, the main factors (monitoring wavelength, chromatographic column, mobile phase, extraction solvent, etc.) that affect the accuracy and sensitivity of eight compounds, including three antioxidants, three light stabilizers, and two plasticizers, were investigated during sample preparation and instrument analysis. A method based on ultrahigh-performance liquid chromatography (UPLC) was developed for the simultaneous determination of these eight additives in polyethylene (PE). The PE food contact material sample was ground to homogenize the particle sizes under freeze-grinding. After comparing the extraction efficiencies of methylbenzene, chloroform, acetone, and acetonitrile, 2.0 g of the sample was extracted with methylbenzene at 80 ℃ and 10.34-11.72 MPa (1500-1700 psi) by accelerated solvent extraction (ASE) for 10 min once. The exaction solvent (10 mL) was transferred and concentrated to near dryness under a gentle stream of nitrogen gas and then re-dissolved in 10 mL of the initial mobile phase (70% (v/v) methanol in water). Finally, the eight compounds were analyzed by UPLC. After optimization of the analytical column and mobile phases, the eight analytes were separated on an ACQUITY UPLC BEH C_8_ chromatographic column (100 mm×2.1 mm, 1.7 μm) by gradient elution using water and acetonitrile as the mobile phases. The column oven temperature, flow rate of the mobile phase, and injection volume were 30 ℃, 0.3 mL/min, and 5 μL, respectively. The analytes were detected by a diode assay detector (DAD) in the scanning range of 210 nm to 400 nm. The monitoring wavelength was set at 230 nm, 250 nm, 280 nm, and 330 nm. External standard calibration curves were used for quantification. Under the optimized conditions, the calibration curves for the eight compounds showed good linearity in the range of 0.2 μg/mL to 10 μg/mL, and the correlation coefficients were >0.999. The recoveries in spiked blank polyethylene samples at the level of 0.05% were in the range of 83.8% to 103.4%, with relative standard deviations (RSDs) ranging from 0.14% to 7.86%. To validate the method, PE reference materials containing these eight compounds were manufactured at the content level of 0.2% to 0.9%. The recoveries using the prepared reference materials ranged from 63.5% to 118.5%, and the RSDs were in the range of 4.61% to 15.6%. The limits of detection (LODs, *S/N*=3) of all the eight compounds were 0.005% and the limits of quantification (LOQs, *S/N*=10) were 0.02%, in compliance with the current legislation. To assess the feasibility and potential of the proposed approach for routine analyses of these eight compounds, the developed method was applied to the analysis of these compounds in ten PE food packages and PE gloves. In six samples, tris(2,4-di-*tert*-butylphenyl)phosphite (Irganox 168) was detected at a level of 0.02%-0.07%, which was lower than the maximum level of this compound in PE food contact material products regulated in GB 9685-2016 at 0.2%. The method is compliant with the current legislation, and it can be used for the monitoring and supervision of these eight additives in PE food contact materials.

聚乙烯(polyethylene, PE)是由乙烯聚合而成的一种热塑性树脂,具有无毒无臭、稳定性好、吸水性小、电绝缘性优良、能耐大多数酸碱等特点,被广泛应用于制造食品包装膜袋,餐厨具与矿泉水瓶等食品接触材料。树脂类食品接触材料在生产和使用过程中易发生氧化裂解,为增强产品性能,会加入抗氧化剂,光稳定剂和增塑剂等添加剂^[1]^。但是在食品接触材料的使用过程中,这些添加剂会迁移至食品中,对人体造成潜在危害^[2,3,4,5]^。鉴于此,我国国家标准GB 9685-2016《食品安全国家标准 食品接触材料及制品用添加剂使用标准》^[6]^以肯定列表的方式规定了允许使用的添加剂品种及最大使用量、残留量、迁移量,欧盟指令(EU)No 10/2011^[7]^也对食品接触材料中迁移有害物的限量进行了规定。因此,建立食品接触材料中添加剂含量及迁移量的测定方法,对于监控食品接触材料的安全性具有重要意义。

目前,食品接触材料中添加剂的检测方法主要为气相色谱法^[8]^、液相色谱法^[9,10]^、气相色谱-质谱法^[11]^,液相色谱-质谱法^[12]^,以及绿色分析方法,如合相色谱、合相色谱-串联四极杆质谱法^[13,14,15,16]^。气相色谱法及气相色谱-质谱法主要针对一些易挥发性组分,如2,6-二叔丁基对苯酚(BHT)、叔丁基对苯二酚(TBHQ)等抗氧剂,邻苯二甲酸酯类增塑剂,二苯甲酮(BP)等光稳定剂的测定^[8]^,应用范围较小。液相色谱及液相色谱-质谱技术的适用范围更广泛,但是大部分文献^[17,18]^只针对食品接触材料中有害物迁移量的测定,对于食品接触材料中有害物含量的测定方法多局限于残留单体、低聚体、重金属^[19,20]^,以及邻苯二甲酸酯类、双酚类化合物等环境污染物^[21,22]^。因此,急需发展食品接触材料中抗氧剂、光稳定剂、增塑剂等添加剂的定量分析方法。

## 1 实验部分

### 1.1 仪器与试剂

Acquity UPLC配二极管阵列检测器(美国Waters公司), 6870D冷冻研磨机(美国SPEX公司), ASE 350加速溶剂萃取仪(美国Thermo公司), MFV-24氮吹仪(广州得泰仪器科技有限公司)。

标准品:亚磷酸三(2,4-二叔丁苯基)酯(抗氧剂168, tris(2,4-di-*tert*-butylphenyl)phosphite, Irganox 168)、邻苯二甲酸二异丁酯(diisobutyl phthalate, DIBP)、邻苯二甲酸双戊酯(diphenyl phthalate, DPP),纯度均≥98%,德国Ehrenstorfer公司;2-(2'-羟基-5'-甲基苯基)苯并三唑(2-(2-hydroxy-5-methylphenyl)benzotriazole, UV-71)、十八烷基-3,5-双(1,1-二甲基乙基)-4-羟基苯丙酸酯(抗氧剂1076, 3,5-bis(1,1-dimethylethyl)-4-hydroxy-benzenepropanoicacioctadecylester, Irganox 1076),纯度≥97%,加拿大TRC公司;2-羟基-4-甲氧基苯并苯酮(2-hydroxy-4-methoxybenzophenone, UV-9),纯度≥99%,北京曼哈格生物科技有限公司;4,4'-硫代双(5-甲基-2-叔丁基苯酚) (抗氧剂300, 4,4'-thiobis(6-*tert*-butyl-*m*-cresol), Irganox 300),纯度≥87%、2-羟基-4-正辛氧基苯并苯酮(2-hydroxy-4-*n*-octyloxybenzophenone, UV-531),纯度≥99%,美国Chemservice公司。甲苯、甲醇、乙腈、丙酮(色谱纯,美国Fisher公司),水为Milli-Q超纯水。

### 1.2 标准溶液的配制

标准储备液:准确称取上述标准品0.1000 g;抗氧剂168、抗氧剂1076、UV-71用丙酮溶解并定容至10 mL,其他5种用甲醇溶解并定容至10 mL,配制成10 g/L的单标准储备液,于-20 ℃保存。

混合标准溶液:根据各化合物的响应,准确移取各标准储备液于10 mL容量瓶中,用甲醇定容,配制成混合标准工作液,再用水-乙腈(3∶7, v/v)逐级稀释,制备系列混合标准溶液。

### 1.3 样品的制备

聚乙烯类食品接触样品用剪刀剪碎至5 mm×5 mm以下,经冷冻研磨至粉末。取2 g样品,采用加速溶剂萃取,甲苯为萃取溶剂,加热温度80 ℃,萃取时间10 min,萃取压力10.34~11.72 MPa(1500~1700 psi);萃取1个循环,收集提取溶剂并定容至200 mL,静置10 min待沉淀析出。取上清10 mL,氮气吹干,用丙酮-甲醇(1∶1, v/v)定容至10 mL。取100 μL,用甲醇-水(7∶3, v/v)定容至1 mL。待上机。

### 1.4 UPLC条件

色谱柱:ACQUITY UPLC BEH C_8_(100 mm×2.1 mm, 1.7 μm);柱温:30 ℃;流动相A:乙腈,流动相B:水;梯度洗脱:见表1;流速:0.3 mL/min;进样量:5 μL; PDA检测波长:210~400 nm。

**表 1:** 色谱分离流动相梯度

No.	Time/min	*φ*(A)/%	*φ*(B)/%
1	0.00	70.0	30.0
2	4.00	70.0	30.0
3	5.00	80.0	20.0
4	6.00	80.0	20.0
5	7.00	100.0	0.0
6	14.00	100.0	0.0
7	15.00	70.0	30.0
8	19.00	70.0	30.0

A: ACN; B: H_2_O.

## 2 结果与讨论

### 2.1 检测波长的确定

8种目标组分混合标准溶液通过液相色谱进样器进样,梯度洗脱后通过二极管阵列检测器在200~400 nm进行扫描分析,得到的二维投影图及每一个化合物的扫描图,8种目标物在低波段有很强的吸收。3种光稳定剂在240、290、330 nm分别有3个较强的吸收峰;2种邻苯二甲酸酯类增塑剂在230和270 nm处有2个较强吸收峰;2种酯类抗氧化剂抗氧剂1076和抗氧剂168在230和270 nm处有2个较强吸收峰;而作为酚类抗氧化剂,抗氧剂300与抗氧剂1076和抗氧剂168明显不同,在250和285 nm处有较强吸收峰。所以本实验采用230、250、280、330 nm作为监测波长,可以同时对8种目标物进行定性和定量。

### 2.2 色谱柱的选择

本研究中的8种目标物极性差别较大,尤其是抗氧剂1076和抗氧剂168属于弱极性化合物,所以选择两种反相色谱柱(C_18_柱和C_8_柱)进行条件优化。结果表明,在优化条件下两种色谱柱均可分离8种目标物。但C_18_色谱柱的保留能力更强,抗氧剂168需要更长的分离时间;而C_8_色谱柱分离时间相对更短,所以本研究采用C_8_色谱柱作为分离柱。在优化后的色谱条件下8种目标物标准溶液的色谱图见图1。

**图 1:**
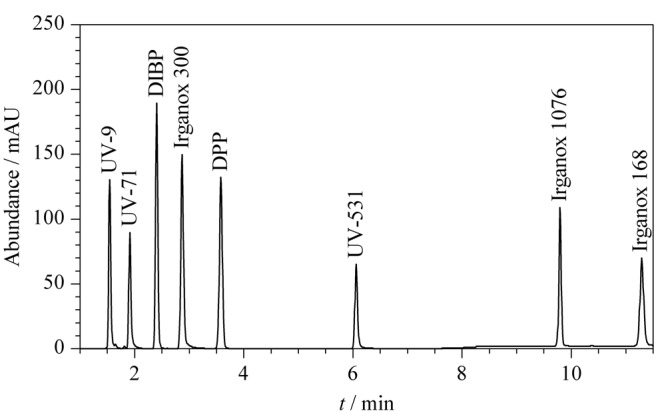
C_8_色谱柱(100 mm×2.1 mm, 1.7 μm)分离 8种目标物的色谱图

### 2.3 流动相的选择

用C_8_色谱柱分离目标物,选择乙腈-水和甲醇-水流动相体系进行优化,两种溶剂体系均能完全分离8种目标物。但是8种目标组分在低波段均有较强吸收,而甲醇的低波段吸收较强,梯度洗脱时低波段的基线漂移较明显,而使用乙腈-水能够避免这种现象。但使用乙腈时,对标准溶液的配制溶剂要求较高,如果直接使用甲醇进行配制,前6种组分出现了前延峰。调整配制溶剂中水的体积分数为30%~50%,峰形得到改善;但水的体积分数在30%以上时,配制50 mg/L以上浓度的混标溶液时出现浑浊,部分化合物析出。所以最后选择乙腈-水溶液作为流动相,标准溶液的配制溶剂为甲醇水溶液(7∶3, v/v)。

### 2.4 样品前处理条件的优化

树脂类食品接触材料属于聚合物,其结构致密,有机试剂的提取效率较低,需要索氏抽提、加速溶剂萃取、超临界流体萃取等方式^[25]^进行提取;另外使用有机溶剂将聚合物溶解或溶胀能够游离出添加成分,从而提高对添加剂提取效率^[26]^。对于聚乙烯类等聚烯烃类食品接触材料,只有使用苯及甲苯类试剂能够将其溶解,艾连峰等^[10]^采用热甲苯进行溶解提取,并用甲醇沉淀聚合物后提取液进行分析,但一次仅能处理0.1 g样品,容易产生样品量小、代表性差导致的检测结果不准确的问题。本研究采用加速溶剂萃取的方式进行前处理,比较了甲苯、氯仿、丙酮、二氯甲烷、乙腈等溶剂,并对加热时间及循环次数进行优化。图2为各种溶剂提取质控样品时的提取效率,可以看出乙腈-二氯甲烷(1∶1, v/v)对各目标物的提取效率最差;对于DIBP、DPP、抗氧剂168,前4种提取溶剂的提取效果较差;只有甲苯作为提取溶剂时,各目标物均有较好的提取效率。进一步对甲苯提取的温度及提取时间进行优化,当加热温度超过100 ℃时,样品有部分溶解,且在提取过程中由于管路中温度低于提取温度,溶解的样品出现了沉淀,造成管路堵塞;采用80 ℃作为提取温度,可获得较好的提取效率,聚合物不会出现溶解再沉淀的现象。对于提取时间,10 min的1个循环提取效率稳定。

**图 2:**
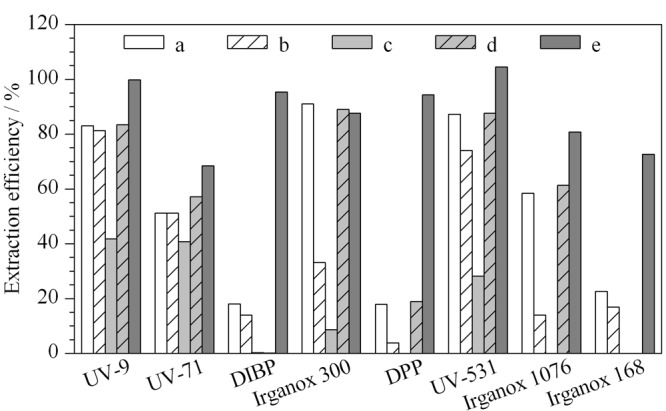
不同溶剂的提取效率

### 2.5 线性关系、检出限和定量限

配制8种目标物的系列混合标准溶液,以目标组分的峰面积*y*对相应的质量浓度*x*(μg/L)绘制标准曲线,结果见表2。结果表明,8种化合物在0.2 ~10 μg/mL之间的线性关系良好,相关系数(*R*^2^)均大于0.99。以*S/N*=3确定方法的检出限,*S/N*=10确定方法的定量限,结果见表2, 8种化合物含量的检出限为0.005%,定量限为0.02%。GB 9685-2016对聚乙烯类食品接触材料中这8种物质的最大使用量要求在0.05%~0.5%之间,所以本方法灵敏度能够满足检测需求。

**表 2:** 8种化合物的线性方程、线性范围、相关系数及方法定量限

Compound	Linear equation	*R* ^2^	LOD/%	LOQ/%
UV-9	*y*=1.76×10^5^*x*-1.60×10^3^	0.9995	0.005	0.02
UV-71	*y*=1.22×10^5^*x*-1.28×10^3^	0.9992	0.005	0.02
DIBP	*y*=2.17×10^5^*x*-7.88×10^2^	0.9994	0.005	0.02
Irganox 300	*y*=2.93×10^5^*x*-2.43×10^3^	0.9995	0.005	0.02
DPP	*y*=2.08×10^5^*x*-1.33×10^3^	0.9995	0.005	0.02
UV-531	*y*=1.01×10^5^*x*-8.50×10^2^	0.9991	0.005	0.02
Irganox 1076	*y*=7.99×10^4^*x*-1.36×10^3^	0.9982	0.005	0.02
Irganox 168	*y*=1.04×10^5^*x*-1.54×10^3^	0.9993	0.005	0.02

*y*: peak area; *x*: mass concentration, mg/L. Linear range: 0.2-10 mg/L.

#### 2.6 方法的准确性

空白PE样品添加含量为0.05%的目标物,进行加标回收试验,8种组分的加标回收率在83.8%~103.4%之间,RSD在0.14%~7.86%之间(见表3)。同时制备质控样品,考察方法的回收率和精密度。模拟聚合物制备流程,采用高速混料机将低密度聚乙烯粉料与8种添加剂混合,混合后的物料采用双螺杆挤出机在高温(190 ℃)熔融状态下挤出造粒(主机转速200 r/min,挤出机转速400 r/min),制备均匀的质控样品聚乙烯颗粒。结果见表4, 8种目标物的平均回收率在63.5%~118.5%之间,RSD在4.61%~15.6%之间。这表明本方法具有较好的准确性。

**表 3:** 聚乙烯空白样品加标含量为0.05%时8种化合物的 回收率和RSD(*n*=6)

Compound	Spiked content/%	Average recovery/%	RSD/%
UV-9	0.05	94.8	2.09
UV-71	0.05	86.9	3.42
DIBP	0.05	103.1	0.14
Irganox 300	0.05	103.4	2.74
DPP	0.05	97.2	7.86
UV-531	0.05	107.7	0.39
Irganox 1076	0.05	105.9	4.94
Irganox 168	0.05	83.8	10.8

**表 4:** 聚乙烯质控样品中8种化合物在3个加标水平下的回收率和RSD(*n*=6)

Compound	Spiked content/%	Average recovery/%	RSD/%
UV-9	0.05	88.4	8.42
	0.2	99.8	4.61
	0.5	92.7	5.02
UV-71	0.1	82.1	13.8
	0.5	94.7	13.4
	0.9	90.1	12.9
DIBP	0.02	74.5	9.13
	0.03	110.3	7.08
	0.05	95.4	4.96
Irganox 300	0.03	82.3	10.6
	0.05	83.8	9.76
	0.09	87.6	6.65
DPP	0.02	82.5	14.3
	0.03	104.7	10.8
	0.05	94.4	4.68
UV-531	0.1	89.5	9.65
	0.5	102.1	4.92
	0.9	118.5	4.87
Irganox 1076	0.1	80.7	10.2
	0.5	89.3	8.96
	0.9	98.3	7.88
Irganox 168	0.05	63.5	15.6
	0.2	64.4	10.9
	0.5	75.6	12.4

#### 2.7 实际样品的测定

采用本方法检测了市售的10份聚乙烯食品包装袋和手套进行测定,其中6份样品均检出抗氧剂168,含量在0.02%~0.07%,均小于GB 9685-2016规定的聚乙烯类食品接触材料中抗氧剂168的最大使用量(0.2%)。典型的色谱图见图3。

**图 3:**
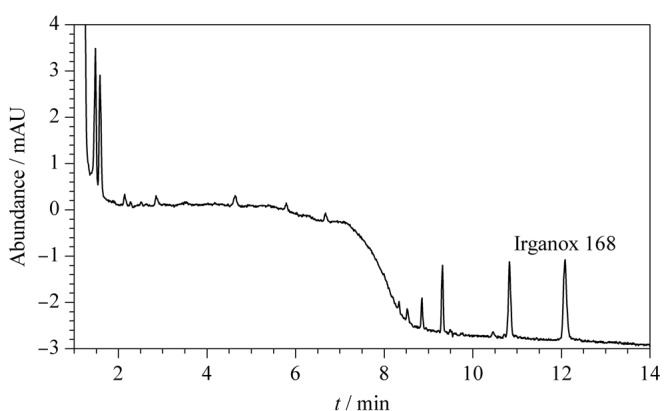
聚乙烯食品接触材料样品的色谱图

## 3 结论

本研究建立了高效液相色谱法同时测定聚乙烯类食品接触材料中8种添加剂的方法,能够处理大量样品,克服了样品量小、代表性差的问题。本研究采用加速溶剂萃取的方式对聚乙烯材料进行快速提取,液相色谱-二极管阵列检测。并采用质控样品考察了方法的准确性,应用于实际样品检测时的重复性良好,能够满足食品接触材料中8种目标物的检测需求,可为食品接触材料中此类化合物的风险监测提供技术支撑。
